# The association of 6 variants of 8q24 and the risk of glioma: A meta-analysis

**DOI:** 10.1097/MD.0000000000016205

**Published:** 2019-07-05

**Authors:** Yu Tong, Lv Ye, Shiping Li, Fengyan Zhao, Junjie Ying, Yi Qu, Jinhui Li, Dezhi Mu

**Affiliations:** a Department of Pediatrics, Key Laboratory of Birth Defects and Related Diseases of Women and Children (Sichuan University), Ministry of Education, West China Second University Hospital, Sichuan University, Chengdu, Sichuan Province; b Chendu Gaoxin-Daan Medical Laboratory Co Ltd Pathology Lab, China.

**Keywords:** 8q24, genetic variant, glioma, meta-analysis, susceptibility

## Abstract

With the advances in sequencing technologies and genome-wide association studies (GWAS), several inherited variants that increase glioma risk have been identified. Ten studies including 8818 cases and 17,551 controls were collected to conduct a meta-analysis to evaluate the associations between 6 variants in 8q24 and glioma risk. Of the 6 variants located in 8q24, 2 have strong significant associations with the risk of glioma, including rs4295627 (*P* = .003, odds ratio [OR] = 1.21), rs55705857 (*P* = 2.31 × 10^–35^, OR = 3.54). In particular, both homozygous GG (*P* = 1.91 × 10^–3^, OR1 = 2.01) and heterozygous GT (*P* = 7.75 × 10^–10^, OR2 = 1.35) genotypes of rs4295627 were associated with glioma risk. Further studies are needed to explore the role of the 8q24 variants involved in the etiology of glioma.

## 1 Introduction

Gliomas account for over 80% of primary malignant brain tumors, however, <50% of glioma patients live longer than 5 years after diagnosis.^[1,2]^ The only established environmental risk factor is exposure to moderate-to-high doses of ionizing radiation.^[3–5]^ With the advances in sequencing technologies and genome-wide association studies (GWAS), several inherited variants that increase glioma risk were firstly been identified.^[6]^ Since then, new risk variants have been identified.^[7–9]^ In 2011, Jenkins et al^[10]^ reported that the glioma risk loci in 8q24 were associated with the development of glioma. These findings were then replicated in different ethnic populations: Safaeian et al^[11]^ followed up on these reports to evaluate the GWAS-identified SNPs on glioma risk in European ancestry, and found that rs4295627 in 8q24 was statistically significantly associated with increased risk of glioma; Wei et al^[12]^ evaluated these glioma risk variants in a Chinese Han population, and found that both the rs891835 and the rs6470745 in 8q24 are associated with increased glioma risk. However, another study did not show any significant associations between 8q24 variants and glioma risk.^[13]^

Several meta-analysis have been done to evaluate the contribution of variants in the 8q24 region to risk of glioma, findings from these studies were generally inconsistent and the mechanisms of these associations remain unclear. Here we performed a comprehensive meta-analysis, involving a total of 8818 cases and 17,551 controls, to evaluate all genetic studies that investigated associations between 6 variants in 8q24 and risk of glioma.

## 2 Methods

### 2.1 Search strategy and selection criteria

We conducted this study in accordance with the guidelines of the Preferred Reporting Items for Systematic Reviews and Meta-Analyses (PRISMA) Statement. We systematically searched PubMed and Embase to identify genetic association studies published in print or online before April 1st, 2018 in English language using key terms “8q24” and “polymorphism or variant or variation or genotype” and “glioma or brain tumor.” Two investigators (YT and JL) independently assessed the eligibility of each study. All studies included in this meta-analysis must meet all the following inclusion criteria: evaluating the associations of the 8q24 variants with glioma risk; providing sufficient data or multivariate-adjusted risk estimates (e.g., odds ratios [ORs], hazard ratios [HRs], relative risks [RRs], 95% confidence intervals (CIs) or standard errors [SEs]) to calculate these estimates. The exclusion criteria were as follows: insufficient data; they were published as letters to editors or conference abstracts; they were studies about cancer mortality.

### 2.2 Data extraction

Guidelines recommended were used to report meta-analyses of observational studies by investigators (YT and JL) to extract data. Extracted data from each eligible study included name of first author, study design, publication data, source population, ethnicity, sample size, variants, alleles, and genotype counts, Hardy–Weinberg equilibrium (HWE) among controls. Ethnicity was classified as Caucasian, African, Asian, or others (such as Latinos, Hawaiians, etc.). In this meta-analysis, 10 eligible publications are available with sufficient data. All analyses were based on previous published studies, thus no ethical approval and patient consent are required.

### 2.3 Statistical analysis and assessment of cumulative evidence

For each study, the OR was used as the metric of choice. Pooled ORs were computed by the fixed effects model and the random effects model based on heterogeneity estimates. Once an overall gene effect was confirmed, the genetic model-free approach suggested by Minelli et al^[14]^ was used to estimate the genetic effects and mode of inheritance. Cochran *Q* test and calculated *І*^2^ statistic were performed to evaluate heterogeneity between studies. *І*^2^ values <25% represent no or little heterogeneity, values 25% to 50% represent moderate heterogeneity, and values >50% represent large heterogeneity. Sensitivity analyses were conducted to examine if exclusion of first published report or studies deviated from HWE in controls influence the significant association. Harbord test was performed to evaluate publication bias. Small study bias was calculated by Egger test. All analyses were conducted using Stata, version 14.0 (StataCorp, Chicago, IL), with the metan, metabias commands.

## 3 Results

### 3.1 Eligible studies:

**Figure 1:**
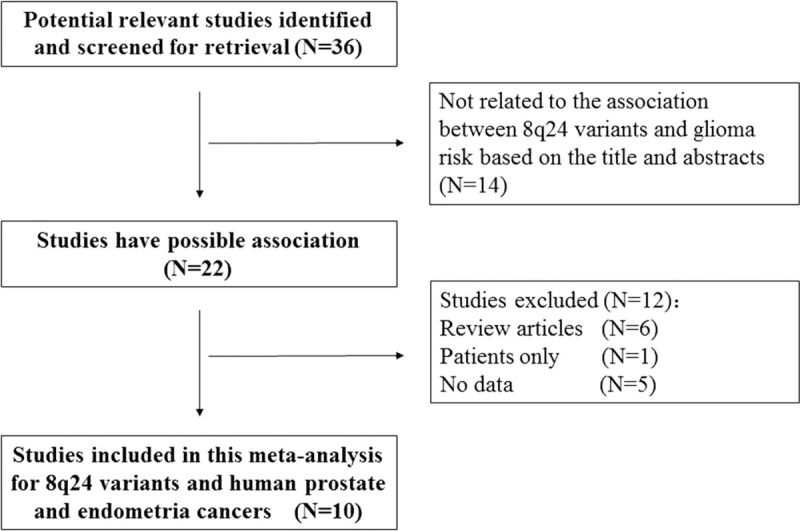
Flow diagram of included and excluded studies.

Our initial database search identified 36 potentially relevant studies. Based on a review of titles and abstracts, 22 publications were retained. The full text of these 22 publications was reviewed in detail, and 10 studies were eligible for inclusion in the meta-analysis. The specific process for identifying eligible studies and inclusion and exclusion criteria are summarized in Fig. 1.

### 3.2 Allelic associations

Of the 6 variants located in 8q24, 2 have strong significant associations with risk of glioma, including rs55705857, rs4295627, and rs6470745 has a weak significant association. No significant associations were found between rs10464870, rs891835 and rs16904140, and glioma (data not shown).

### 3.3 rs4295627 T>G

**Figure 2:**
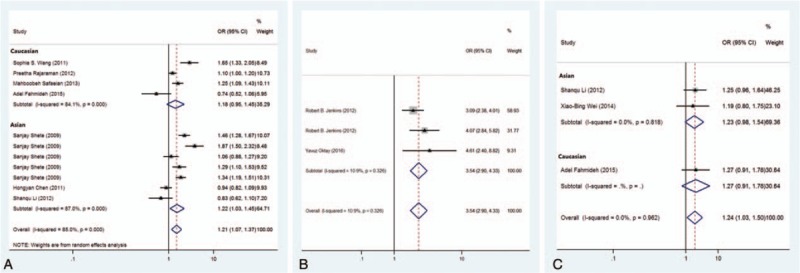
Forest plots for associations between selected variants in the 8q24 region and glioma risk. Associations of rs4295627 (A), rs55705857 (B), rs6470745 (C) with glioma risk.

Seven studies were included (Table 1), and a significant association with risk of glioma was found (*P* = .003, random effect OR = 1.21, 95% CI: 1.07, 1.37; *Q* = 66.78, *P* = .000, *I*^2^ = 85.0%, Fig. 2A). A similar pattern was observed for Asians (*P* = .022, random effect OR = 1.22, 95% CI: 1.03, 1.46; *Q* = 46.1, *P* = .000, *I*^2^ = 87.0%). No publication bias was found in the eligible studies (Harbord test *P* = .990, Table 2).

**Table 1:**
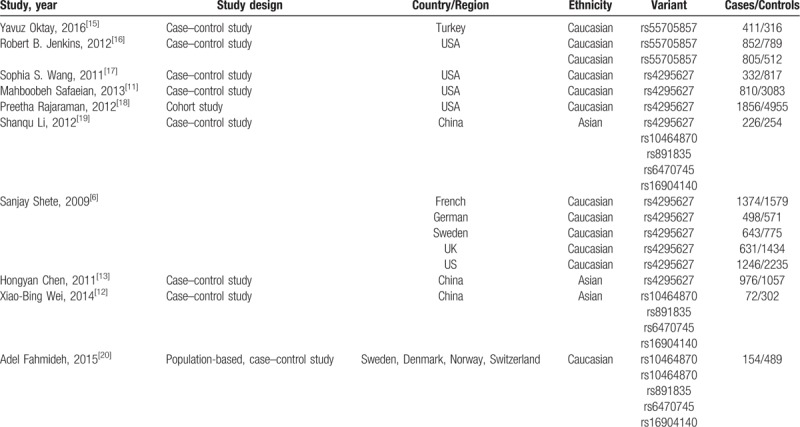
Characteristics of the included articles.

**Table 2:**
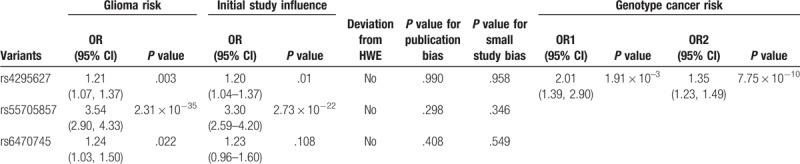
Details of genetic variants significantly associated with gliomas in meta-analyses.

### 3.4 rs55705857 A>G

Three studies were included (Table 1), and a significant association with risk of glioma was found (*P* = 2.31 × 10^–35^, fixed effect OR = 3.54, 95% CI: 2.90, 4.33; *Q* = 2.24, *P* = .326, *I*^2^ = 10.9%, Fig. 2B). No publication bias was found in the eligible studies (Harbord test *P* = .298, Table 2).

### 3.5 rs6470745 A>G

Three studies were included (Table 1), and a significant association with risk of glioma was found (*P* = .022, fixed effect OR = 1.243, 95% CI: 1.032, 1.498; *Q* = 0.08, *P* = .962, *I*^2^ = 0.0%, Fig. 2C). No publication bias was found in the eligible studies (Harbord test *P* = .408, Table 2).

### 3.6 Genotype comparison

Only 3 studies reported the genotype information of rs4295627 T>G. The genotype effects for GG versus TT (OR1) and GT versus TT (OR2) were calculated for each study. A multivariate meta-analysis was conducted to estimate the pooled risk (Table 2). There was a significantly increased risk of glioma among individuals with the homozygous GG genotype (*P* = 1.91 × 10^–3^, random effect OR1 = 2.01, 95% CI: 1.39, 2.90; *Q* = 25.29, *P* = .000, *I*^2^ = 76.3%) and heterozygous GT genotype (*P* = 7.75 × 10^–10^, random effect OR2 = 1.35, 95% CI: 1.23, 1.49; *Q* = 9.92, *P* = .128, *I*^2^ = 39.5%).

### 3.7 Sensitivity analysis

Sensitivity analysis for the results of 8q24 variants and glioma risk demonstrated that the obtained results were statistically robust (Table 2).

## 4 Discussion

To our knowledge, this is the first general overview of the association between 8q24 variants and susceptibility to glioma. Preliminary meta-analyses were mostly limited to single or less SNPs in relation to glioma. Here we performed a research synopsis and meta-analysis to systematically evaluate associations between 6 variants in 8q24 region and glioma risk using data from 22 publications totaling 8818 cases and 17,551 controls. Our study not only provides an update of the previously analyzed variants, but also evaluates more other variants that have not been analyzed by meta-analyses previously.

Of the 6 variants located in 8q24, 2 have strong significant association with risk of glioma, 1 has a weak significant association with risk of glioma. Our primary analysis shows that, the rs4295627 (*P* = .003, OR = 1.21), rs55705857 (*P* = 2.31 × 10^–35^, OR = 3.54) have strong significant associations with the risk of glioma. In particular, both homozygous GG (*P* = 1.91 × 10^–3^, OR1 = 2.01) and heterozygous GT (*P* = 7.75 × 10^–10^, OR2 = 1.35) genotypes of rs4295627 were associated with glioma risk. Based on several gene-association studies and several thousand participants, our findings were robust in terms of study design and sensitivity analyses. No evidence of publication bias or small study bias was found.

Several preliminary meta-analyses focused on evaluating association between a single SNP (rs295627) in the 8q24 region and risk of glioma.^[18,21]^ No systematic research synopsis has been published to evaluate cumulative evidence of genetic associations of 8q24 with glioma thus far. Newly published studies should be included so as to increase the statistical power and generate more precise and updated results. In this study, we attempted to evaluate all genetic studies that investigated associations between variants in the 8q24 region and risk glioma, and we performed meta-analyses for variants with available sufficient data and summarized cumulative epidemiological evidence for variants with significant association.

Both the rs4295627 and the rs55705857 are mapped to the *CCDC26*. Genetic association studies, especially GWAS, indicated that the rs4295627 SNP may contribute to the increased risk of glioma. The rs55705857 G-allele can also increase tumorigenic potential of tumor initiating cells in oligodendrogliomas and astrocytomas. The differential activity of Ras/MAPK pathways and the RTK activation are involved in this process.^[15]^ Some research has indicated that the expression of CCDC26 upregulated in glioma tissues and cell lines, and CCDC26-siRNA inhibited glioma growth and metastasis.^[22]^ Further studies are needed to clarify the physiological function of CCDC26 and the functional consequence of these variants in vivo.

There are several limitations of the study should be mentioned. First, it is likely that some publications were overlooked although we conducted an exhaustive literature search, some relevant published studies or unpublished studies with null results were not identified. Second, data about the types of glioma was absent from most of the 22 studies included in this meta-analysis. Third, due to insufficient data, we were unable to evaluate publication bias for associations between several variants in 8q24 region and glioma. Therefore, future studies with larger sample size are warranted to confirm these associations.

In summary, we have identified 3 variants in the 8q24 region that showed strong evidence of an association with glioma risk in this large-scale research synopsis and meta-analysis. Further functional studies are needed to explore the exact mechanisms of 8q24 variants involved in parthenogenesis of glioma.

## Author contributions

**Data curation:** Yu Tong.

**Formal analysis:** Yu Tong.

**Investigation:** Fengyan Zhao.

**Project administration:** Dezhi Mu.

**Software:** Lv Ye, Shiping Li, Junjie Ying.

**Supervision:** Yi Qu, Jinhui Li.

**Writing – original draft:** Yu Tong.

**Writing – review & editing:** Dezhi Mu.

Dezhi Mu orcid: 0000-0002-2599-7041.
